# Effect of a two-stage intervention package on the cesarean section rate in Guangzhou, China: A before-and-after study

**DOI:** 10.1371/journal.pmed.1002846

**Published:** 2019-07-08

**Authors:** Xiaoyan Xia, Zehong Zhou, Songying Shen, Jinhua Lu, Lifang Zhang, Peiyuan Huang, Jia Yu, Li Yang, Ping Wang, Kin-bong Hubert Lam, Bo Jacobsson, Ben Willem Mol, Huimin Xia, Xiu Qiu

**Affiliations:** 1 Division of Birth Cohort Study, Guangzhou Women and Children’s Medical Center, Guangzhou Medical University, Guangzhou, China; 2 Department of Woman and Child Health Care, Guangzhou Women and Children’s Medical Center, Guangzhou Medical University, Guangzhou, China; 3 Department of Obstetrics and Gynecology, Guangzhou Women and Children’s Medical Center, Guangzhou Medical University, Guangzhou, China; 4 Guangzhou Women and Children’s Health Information Center, Guangzhou, China; 5 Nuffield Department of Population Health, University of Oxford, Oxford, United Kingdom; 6 Department of Obstetrics and Gynecology, Sahlgrenska Academy, University of Gothenburg, Gothenburg, Sweden; 7 Department of Genetics and Bioinformatics, Domain of Health Data and Digitalisation, Institute of Public Health, Oslo, Norway; 8 Department of Obstetrics and Gynecology, Monash University, Clayton, Victoria, Australia; 9 Department of Obstetrics and Gynecology, University of Birmingham, Birmingham, United Kingdom; 10 Department of Neonatal Surgery, Guangzhou Women and Children’s Medical Center, Guangzhou Medical University, Guangzhou, China; King's College London, UNITED KINGDOM

## Abstract

**Background:**

The cesarean section (CS) rate has risen globally during the last two decades. Effective and feasible strategies are needed to reduce it. The aim of this study was to assess the CS rate change after a two-stage intervention package that was designed to reduce the overall CS rate in Guangzhou, China.

**Methods and findings:**

This intervention package was implemented by the Health Commission of Guangzhou Municipality in 2 stages (October 2010–September 2014 and October 2014–December 2016) and included programs for population health education, skills training for healthcare professionals, equipment and technical support for local healthcare facilities, and capacity building for the maternal near-miss care system. A retrospective repeated cross-sectional study was conducted to evaluate influences of the intervention on CS rates. A pre-intervention period from January 2008 to September 2010 served as the baseline. The primary outcome was the CS rate, and the secondary outcomes included maternal mortality ratio (MMR) and perinatal mortality rate (PMR), all obtained from the Guangzhou Perinatal Health Care and Delivery Surveillance System (GPHCDSS). The Cochran-Armitage test was used to examine the trends of the overall CS rate, MMR, and PMR across different stages. Segmented linear regression analysis was used to assess the change of the CS rate over the intervention period.

A total of 1,921,932 records of births and 108 monthly CS rates from 2008 to 2016 were analyzed. The monthly CS rate declined across the intervention stages (Z = 75.067, *p* < 0.001), with an average rate of 42.4% at baseline, 39.8% at Stage 1, and 35.0% at Stage 2. The CS rate declined substantially among nulliparous women who delivered term singletons, with an accelerating decreasing trend observed across Stage 1 and Stage 2 (the difference in slopes: −0.09 [95% CI −0.16 to −0.02] between Stage 1 and baseline, *p* = 0.014; −0.11 [95% CI −0.20 to −0.02] between Stage 1 and Stage 2, *p* = 0.017). The CS rate in the remaining population increased during baseline and Stage 1 and subsequently decreased during Stage 2. The sensitivity analysis suggested no immediate impact of the universal two-child policy on the trend of the CS rate. The MMR (*Z* = −4.368, *p* < 0.001) and PMR (*Z* = −13.142, *p* < 0.001) declined by stage over the intervention period.

One of the main limitations of the study is the lack of a parallel control group. Moreover, the influence of temporal changes in the study population on the CS rate was unknown. Given the observational nature of the present study, causality cannot be confirmed.

**Conclusions:**

Apparent decline in the overall CS rate was observed in Guangzhou, China, after the implementation of a two-stage intervention package. The decline was most evident among nulliparous women who delivered term singletons. Despite some limitations for causal inference, Guangzhou’s experience in controlling the CS rate by implementing composite interventions with public health education and perinatal healthcare service improvement could have implications for other similar areas with high rates of CS.

## Abstract 2

Xiu Qiu and colleagues report on the cesarean section rate in Guangzhou, China following implementation of a two-stage intervention package.

## Author summary

**Why was this study done?:**

- Despite the association between cesarean section (CS) and a wide range of adverse maternal and neonatal outcomes, a nationwide study in China reported an important public health problem, with the CS rate rising from 28.8% in 2008 to 34.9% in 2014.
- Evaluation of the effectiveness and feasibility of interventions for reducing CS rate is needed to provide implications for other settings with similar high rates.

**What did the researchers do and find?:**

- We retrospectively evaluated the change of the CS rate before and after a two-stage intervention package consisting of strategies for public health education and perinatal healthcare service improvement. It was implemented by the government of Guangzhou, China, from October 2010 to December 2016 to control the CS rate in the city.
- Data of 1,921,932 births between 2008 and 2016 were obtained from a city-wide health surveillance system.
- The average CS rate in all births decreased from 42.4% at baseline (Jan 2008–Sep 2010) to 39.8% at Stage 1 (October 2010–September 2014) and to 35.0% at Stage 2 (October 2014–December 2016), with nulliparous singletons at term largely accounting for the decline.
- Along with the decreasing CS rate, maternal and perinatal mortality also declined across different stages.

**What do these findings mean?:**

- Comprehensive intervention strategies reported in this study could safely reduce the CS rate, providing valuable experience for other areas with similar high CS rates.
- Nulliparous women should be the target population for the control of CS rate.
- Given the lack of comparable groups and the temporal change in the study population, the contribution of this intervention package should be interpreted with caution.

## Introduction

Cesarean section (CS), although essential and lifesaving with the correct indication, is largely unnecessary in nonemergency conditions, leading to greater financial costs relative to vaginal delivery, without proven benefit, and increasing risks for maternal morbidity and mortality [1–8]. In addition, CS has been associated with elevated risks of adverse maternal and perinatal outcomes in future pregnancies, including maternal and intra-uterine fetal death [9–13].

Since 1985, the World Health Organization (WHO) has recommended that national CS rates not exceed 10% to 15% [14]. A statement issued in 2016 further strengthened WHO’s position, with evidence suggesting that population-level CS rates higher than 10% are not associated with reduced maternal and neonatal mortality [15]. Contrary to the recommendation, the rate of cesarean delivery has been rising sharply in the last two decades worldwide [16,17]. A study of 194 WHO member states estimated global cesarean deliveries to be 22.9 million in 2012 [16]. A nationwide study covering 2,865 counties in China reported that the overall annual CS rate rose from 28.8% in 2008 to 34.9% in 2014 [18]. Interestingly, data from 438 Chinese hospitals in the National Maternal Near Miss Surveillance System showed that the CS rate declined from 45.3% in 2012 to 41.1% in 2016 [19]. In Guangzhou, the third largest city in China with a population of 14 million and 300,000 annual births, the CS rate was about 40% between 2001 and 2010, far higher than the national average and the level recommended by WHO.

The risk for adverse outcomes has prompted the Health Commission of Guangzhou Municipality to investigate strategies to reduce CS rates and to improve the quality of intrapartum care [18]. A review was carried out and identified a number of strategies shown to be effective in a range of settings [20–22]. Although the drivers of high CS rates in Guangzhou have not been investigated, other areas of China have reported that CS rates may have been driven up because of fear of pain during labor and of perceived risks for the baby caused by vaginal birth, distrust of and dissatisfaction with the healthcare system, and intrinsic cultural beliefs popular among Chinese families [23,24]. Based on these findings, the Health Commission determined that the most appropriate intervention should aim to raise public knowledge and awareness towards CS and to improve the quality of maternal healthcare services. Consequently, the Action Plan for Safe Motherhood and Infancy, a comprehensive health promotion program aiming to control and reduce maternal and infant mortality in Guangzhou, was developed in 2010. As an integral component of this plan, an intervention package intended to reduce CS rate was implemented in 2 stages (October 2010 to September 2014 and October 2014 to December 2016) and comprised programs for population health education, skills training to healthcare professionals, equipment and technical support for local healthcare facilities, and capacity building for the maternal near-miss care system.

Using routine surveillance data between January 1, 2008, and December 31, 2016, the present study aimed to evaluate changes of the overall CS rate in Guangzhou over the period of the two-stage intervention. Trends in maternal and perinatal mortality were also examined to evaluate the safety of the intervention package when reducing the CS rate.

## Materials and methods

### Study design

This is a repeated cross-sectional study aiming to retrospectively evaluate a two-stage intervention package implemented by the municipal government to reduce the overall CS rate in Guangzhou. The conduct of this study was fully compliant with relevant national regulations and was approved by the institutional ethical committee board of Guangzhou Women and Children’s Medical Center. An analysis plan was developed prior to the study (S1 File). This study is reported as per the REporting of studies Conducted using Observational Routinely-collected health Data (RECORD) guideline (S1 Checklist).

### Design and implementation of the two-stage intervention package

The intervention was part of a large-scale municipality-wide maternal and neonatal health promotion program, the Action Plan for Safe Motherhood and Infancy, with the overall aim to reduce maternal and neonatal mortality in Guangzhou. Detailed information about the intervention package is presented in Table 1. Before the intervention began, detailed documentation on the contents, requirements, targets, timeline, responsibilities, funding, supervision, quality assurance, and evaluation approaches was issued to all relevant government agencies and healthcare facilities in Guangzhou. There were 3 elements in Stage 1 (October 1, 2010, to September 30, 2014): health education, skills training, and capacity building. Full-time staff were appointed to take up the responsibility of health education in all 12 districts of Guangzhou, organizing lectures for pregnant women and their family members on the advantages of vaginal delivery as well as other important topics in perinatal health. Also as part of the publicity campaign, they held roadshows in the community with posters and videos and handed out information leaflets. For the skills training element, specialists from secondary and tertiary hospitals (larger in scale and more specialized in healthcare services) were paired up with local primary facilities to provide intensive clinical training for midwives and obstetricians, focusing on pain-free vaginal delivery and other advanced clinical management strategies and techniques. For capacity building, 6 new municipal maternal near-miss care centers were set up to fill the void in the maternal and child healthcare and referral system in Guangzhou (S1 Fig). Towards the end of Stage 1, an interim evaluation and a comprehensive needs assessment for the public, for healthcare institutions, and for public health agencies were conducted. Having incorporated input from stakeholders, the municipal government launched the second intervention stage (October 1, 2014, to December 31, 2016). The health education and skills training elements were maintained with minor adjustments. Resources were allocated to provide equipment and technical support for healthcare facilities and to construct 8 district-level care centers for maternal near misses in this stage.

**Table 1:** Strategies of the two-stage intervention for reducing CS rates in Guangzhou, China, 2010–2016.

Intervention Elements	Stage 1 (October 1, 2010–September 30, 2014)		Stage 2 (October 1, 2014–December 31, 2016)	
	Plans and Strategies	Outputs	Plans and Strategies	Outputs
Health education	(1) Professional training	(1) 125 full-time health education professionals trained, covering all 12 districts	(1) Professional training	(1) 233 full-time health education professionals trained, covering all 12 districts
	● 1 full d/session, 4 sessions/person		● Duration, frequency, and target skills: same as Stage 1	
	● Target skills: communication, presentation, community-level health education methodology		● Training at least 1 professional from each maternity facility	
	(2) Community lecturing	(2) 100 lectures delivered, covering all 12 districts	(2) Community lecturing	(2) 100 lectures delivered, covering all 12 districts
	● Target subjects: pregnant women and their families		● Target subjects, duration, coverage, and main topics: same as Stage 1	
	● 90 min/lecture		● Additional topics: labor analgesia, maternal mental health	
	● Main topics: pregnancy planning, advantages of vaginal delivery, maternal nutrition and exercise, postpartum care for vaginal and cesarean delivery			
	(3) Publicity platform building	(3) Leaflets and presenting posters disseminated in all communities and maternity facilities, videos played on approximately 40% of the buses across the city	(3) Publicity platform building	(3) Leaflets, posters, and videos: same as Stage 1; publicity videos played on local TV channels and outdoor LED screens across the city, educational articles posted on local newspapers and social media
	● Cooperating with professional and designers		● Production, approaches, and main topics: same as Stage 1	
	● Including leaflets, posters, videos		● Additionally including multimedia approaches	
	● Main topics: vaginal birth, routine prenatal care, maternal lifestyles during pregnancy, postpartum care			
			(4) “Toolbox” production	(4) A “toolbox” produced, containing 800+ collated and reorganized items of materials, with full access open to all health education professionals
			● For creating new health education materials	
			● Including a collection of relevant articles, leaflets, posters, videos	
			● Including raw materials (e.g., slide templates, pictures, footage, etc.)	
			● Covering main topics in (2) and (3)	
Skills training	(1) Expert on-site instructions	(1) Experts assigned to 82 primary and secondary healthcare institutions, each expert working on site for 100 d	(1) Core skills training	(1) 100 obstetricians, 100 midwives, 100 pediatricians, and 100 pediatric nurses trained
	● Target subjects: obstetricians, anesthesiologists, and midwives from primary and secondary healthcare institutions		● Target subjects: obstetricians, midwives, pediatricians, and pediatric nurses from primary and secondary healthcare institutions	
	● Target skills: up-to-date clinical knowledge and skills		● Target skills: the same core skillset for obstetrics and gynecology, pediatrics, maternal and child health at Stage 1	
	● Approaches: teaching, thematic training, discussion of challenging cases, and surgical demonstration		● Off-the-job training for 2 mo in designated training centers with exit exams	
	(2) Core skills training	(2) 200 obstetricians, 100 neonatologists, and 100 midwives trained	(2) Advanced skills training	(2) Training coverage of 100% in all maternity facilities
	● Target subjects: backbones of obstetricians, neonatologists, and midwives from primary healthcare institutions		● Target subjects and skills: same as Stage 1	
	● Target skills: core knowledge and skills in perinatal practice (e.g., analgesic vaginal delivery, labor process monitoring using partogram, etc., with the emphasis of reducing unnecessary clinical interventions)			
	● Off-the-job training for 3–6 mo in designated tertiary hospitals with exit exams			
	(3) Advanced skills training	(3) Training coverage of 100% in all primary and secondary healthcare institutions		
	● Target subjects: obstetric, pediatric, midwifery, and anesthesiologic professionals from primary and secondary healthcare institutions			
	● Target skills: pediatric advanced life support			
Equipment and technical support for primary and secondary maternity facilities	Not applicable	Not applicable	(1) Basic equipment support	(1) 68 primary and secondary maternity facilities supported, covering all 12 districts
			● Target departments: obstetric and pediatric outpatient, emergency, inpatient, ICU, and medical laboratory departments	
			(2) Specific technical support	(2) Experts assigned to 10 selected maternity facilities, each expert working on site for 3 mo
			● Expert in-site instructions on specific techniques	
			● Target institutions: selected secondary maternity facilities based on demands and existing resources	
			● Target techniques: vital sign monitoring, forceps delivery, and neonatal resuscitation	
Capacity building for the maternal near-miss care system	(1) Capacity expansion for municipal-level maternal near-miss care centers	(1) 6 new municipal-level maternal near-miss care centers established for specific diseases	(1) Capacity building for district-level maternal near-miss care centers	(1) 8 new district-level maternal near-miss care centers established; a long-term municipal-district twinned support system set up
	● From “one for all” to “specialized maternity intensive care”		● Increasing the number of centers based on the demand of district	
			● Developing municipal-district twined support to enhance technical cooperation	

Information in this table was obtained from the proposals and annual reports of the Action Plan for Safe Motherhood and Infancy.

**Abbreviations:** CS, cesarean section; ICU, intensive care unit; LED, light emitting diode

### Data collection

For the purpose of this study, we defined a pre-intervention period between January 1, 2008, and September 30, 2010, as the baseline. Computerized birth data between 2008 and 2016 were obtained from Guangzhou Perinatal Health Care and Delivery Surveillance System (GPHCDSS), which has been described elsewhere [25–28]. Briefly, the GPHCDSS was initiated in 2000 and covers over 99% of deliveries in Guangzhou. Within the surveillance system, birth information of neonates born in hospitals is reported to the Health Commission of Guangzhou Municipality via a computer network and is used to issue birth certificates. Dedicated trained health workers are responsible for collating and registering birth information at each hospital. Validity of the data is confirmed by the chief midwife and the chief physician in the hospital after data entry. The Department of Medical Administration and the newborn’s parents also validate the information when the birth certificate is issued. The Health Commission verifies the GPHCDSS data annually through sampling survey to ensure data reliability. Data from the GPHCDSS do not contain individually identifiable information, and therefore individual informed consent was not required. We only included births delivered at gestational age of ≥28 weeks. From the GPHCDSS, we collected information on maternal age, gravidity, parity, gestational age, birth weight, and mode and outcomes of delivery. Data on maternal deaths were collected through the Maternal and Child Health Data Reporting Network in China [29]. We excluded observations with extreme values (e.g., parity and gravidity above the 99.98 percentile or women aged <14 years) or missing gestational age information.

### Outcomes

The primary outcome was the overall CS rate, defined as the number of cesarean deliveries per 100 births. CS rates were calculated for each month and for each of the 3 stages (baseline, Stage 1, and Stage 2). The monthly CS rates were directly age standardized using the 2008 population in each group in the study. The secondary outcomes were the maternal mortality ratio (MMR), defined as the number of maternal deaths (during pregnancy or within 42 days of termination of pregnancy, except for accidental deaths) per 100,000 live births, and the perinatal mortality rate (PMR), defined as the number of stillbirths (≥28 weeks of gestation) and early neonatal deaths (0–6 days) per 1,000 births [18]. Women with a diagnosis of uterine scarring along with a history of delivery were considered to have a prior CS. Those having multiparous births but without a uterine scarring diagnosis were assumed to have a prior vaginal delivery.

### Statistical analysis

Average CS rate was calculated among all births and cross-tabulated with maternal and perinatal characteristics (maternal age: <25, 25–29, 30–34, and ≥35 years; parity: nulliparous, multiparous with prior vaginal delivery, and multiparous with prior CS; gestational age: preterm [<37 weeks], term [37–42 weeks], and post-term [>42 weeks]; and birth weight: low birth weight [<2,500 g], normal birth weight [2,500–3,999 g], and macrosomia [≥4,000 g] for each of the 3 intervention stages, respectively). The Cochran-Armitage test was used to examine the changing trends of overall CS rates, MMR, and PMR before and after intervention. Segmented linear regression analysis of interrupted time series [30,31] was used to examine changes in age-standardized rates (ASRs) of CS per month over the period of the two-stage intervention, stratified into nulliparous singletons at term and other births. Durbin-Watson tests were used to check for autocorrelation; those statistic values close to 2.0 indicated that there was no serious autocorrelation. For monthly data, stepwise autoregression was used to select the autoregressive error model automatically, and subset models for season monthly data were conducted if needed. A sensitivity analysis excluding the last 3 months in 2016 was conducted to assess the impact of the universal two-child policy (implemented in October 2015) on CS rate. To understand the change in CS rates over time at the institutional level, 112 hospitals that were part of the midwifery system in Guangzhou during 2008–2016, with more than 100 live births at both baseline and in Stage 2, were grouped according to their baseline CS rates (<30%, 30%–39%, 40%–49%, and ≥50%), and both the absolute and the relative decrease in CS rate at individual hospitals from baseline to Stage 2 were depicted on a scatter plot. One-way analysis of variance compared the decrease in CS rates across different groups of hospitals. To clarify the change of fetal presentation on cesarean deliveries, we analyzed breech and shoulder presentation and cephalic presentation separately stratifying for parity and prior delivery mode (nulliparous singletons at term, prior vaginal delivery, and prior CS). Analyses were performed using SAS version 9.3 (SAS Institute, Cary, NC) and SPSS version 23.0 (SPSS, Chicago, IL). All *p-*values were two-sided, and *p* < 0.05 was considered to indicate statistical significance.

## Results

### Study population

A total of 1,923,687 records of babies born ≥28 gestational weeks between January 1, 2008, and December 31, 2016, were retrieved, of which 1,755 were excluded due to missing or implausible data on parity (*n* = 507), gravidity (*n* = 524), and gestational age (*n* = 670) or maternal age (<14 years, *n* = 54). As a result, 1,921,932 births and 108 monthly CS rates were included in the analysis.

### Trends in maternal and perinatal characteristics

Maternal and perinatal characteristics by stage are shown in Table 2 and S2 Fig. The proportion of births to mothers of advanced age (≥35 years) increased gradually from 9% at baseline to 10% at Stage 1, and to 14% at Stage 2. Over the intervention period, the incidence of preterm birth increased slightly from 7% at baseline to 8% at Stage 2, while the birth weight decreased from 3,159 g to 3,148 g. Maternal and perinatal characteristics of nulliparous singletons at term and other births are shown in S1 Table and S2 Table, respectively.

**Table 2:** Perinatal characteristics and CS rates by intervention stages among all births in Guangzhou, China, 2008–2016 (*n* = 1,921,932).

Variable	Perinatal characteristics, *N* (%)			CS rates, % (95% CI)			
	Baseline	Stage 1	Stage 2	Total	Baseline	Stage 1	Stage 2
**Total**	461,985 (24.0)	902,147 (46.9)	557,800 (29.0)	39.0 (38.4–39.6)	42.4 (42.0–42.7)	39.8 (39.3–40.2)	35.0 (34.4–35.5)
**Maternal age (y) (*N* = 1,914,289)**							
Mean (SD)	27.9 (4.8)	28.4 (4.9)	29.4 (5.0)				
<25	134 006 (29)	232 183 (26)	104 172 (19)	28.9 (28.1–29.6)	32.9 (32.5–33.3)	29.5 (29.0–30.0)	22.3 (21.7–22.9)
25–29	187 168 (41)	361 428 (40)	220 117 (39)	36.7 (35.8–37.7)	42.3 (41.8–42.7)	37.8 (37.2–38.4)	30.2 (29.6–30.8)
30–34	97 645 (21)	215 787 (24)	156 077 (28)	45.6 (44.8–46.3)	49.5 (49.0–50.1)	46.9 (46.3–47.5)	41.2 (40.4–41.9)
≥35	40 340 (9)	87 948 (10)	77 418 (14)	55.9 (55.3–56.5)	57.9 (57.3–58.5)	57.4 (56.9–57.9)	53.2 (52.5–53.9)
**Parity (*N* = 1,916,508)**							
Nulliparas	299,722 (65)	513,848 (57)	269,952 (49)	39.2 (38.1–40.3)	44.9 (44.5–45.4)	40.3 (39.6–41.0)	30.7 (30.0–31.4)
Multiparas with PCS	28,515 (6)	90,068 (10)	80,502 (14)	92.6 (92.1–93.1)	94.5 (94.1–94.9)	94.2 (93.9–94.5)	90.2 (89.7–90.7)
Multiparas with PVD	132,820 (29)	296,823 (33)	204,258 (37)	21.9 (21.4–22.5)	25.3 (24.9–25.8)	22.3 (21.9–22.7)	19.1 (18.4–19.8)
**Gestational weeks (*N* = 1,921,932)**							
Preterm	32,457 (7)	68,048 (8)	43,111 (8)	50.2 (49.5–51.0)	45.8 (45.0–46.7)	50.8 (49.8–51.8)	52.6 (51.7–53.6)
Term	428,739 (93)	833,508 (92)	514,581 (92)	38.1 (37.4–38.8)	42.1 (41.7–42.4)	38.9 (38.4–39.3)	33.5 (33.0–34.0)
Post-term	789 (0.2)	591 (0.1)	108 (0.02)	49.5 (47.2–51.9)	52.3 (49.6–55.1)	48.2 (44.1–52.4)	36.1 (24.8–47.4)
**Birth weight (*N* = 1,921,039)**							
Mean (SD) (g)	3,159 (478)	3,153 (475)	3,148 (476)				
Low birth weight	28,465 (6)	58,681 (7)	37,907 (7)	51.7 (51.0–52.4)	47.2 (46.3–48.0)	52.5 (51.5–53.5)	53.9 (52.8–54.9)
Normal	416,835 (90)	814,159 (90)	502,375 (90)	37.4 (36.7–38.0)	41.2 (40.8–41.6)	38.1 (37.7–38.6)	33.0 (32.4–33.5)
Macrosomia	16,452 (4)	29,100 (3)	17,065 (3)	59.2 (58.3–60.1)	63.2 (62.3–64.2)	60.1 (59.2–61.0)	53.8 (52.8–54.9)

Baseline: January 2008–September 2010; Stage 1: October 2010–September 2014; Stage 2: October 2014–December 2016.

**Abbreviations:** CS, cesarean section; PCS, prior cesarean section; PVD, prior vaginal delivery

### Trends in CS rates on the population level

**Fig 1:**
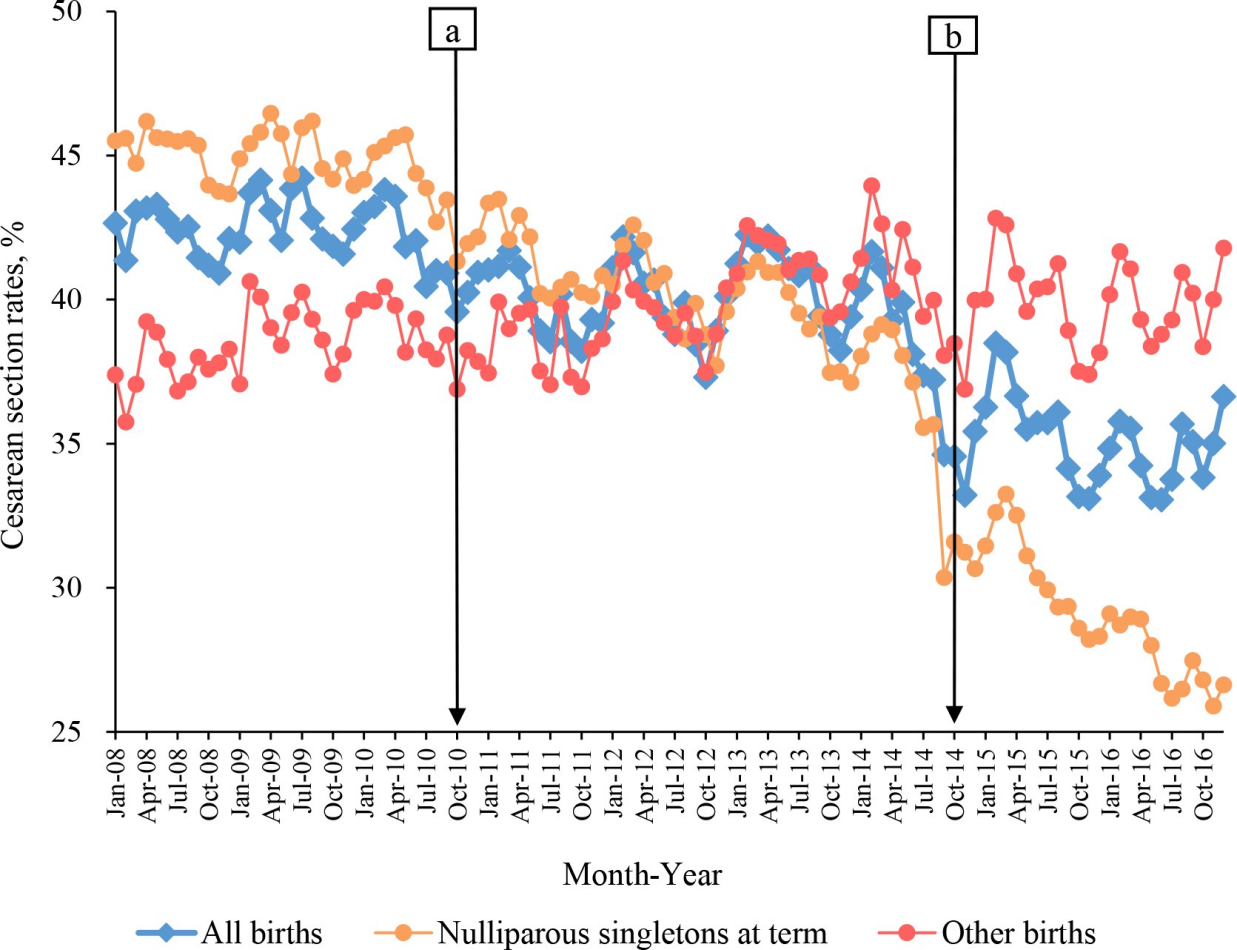
The trend of CS rates in all births, nulliparous singletons at term, and other births in Guangzhou, China, 2008–2016. Blue circles: CS rates for all births; orange: CS rates for nulliparous singletons at term; red: other births. Baseline: from January 2008 to September 2010; Stage 1: from October 2010 (Point “a”) to September 2014; Stage 2: from October 2014 (Point “b”) to December 2016. CS, cesarean section.

Fig 1 shows the secular trend of CS rates in all births and in subgroups of nulliparous singletons at term and other births. Overall, there was a decreasing trend in the monthly CS rates over the 3 periods (*Z* = 75.067, *p* < 0.001), with 42.4% (95% CI 42.0%–42.7%) at baseline, 39.8% (39.3%–40.2%) at Stage 1, and 35.0% (34.4%–35.5%) at Stage 2 (Table 2, Fig 1). The apparent decrease among nulliparous singletons at term (*Z* = 116.683, *p* < 0.001) largely accounted for the overall declining trend observed in all births. By contrast, there was a gradual increase in CS rates among other births (*Z* = −7.359, *p* < 0.001).

**Fig 2:**
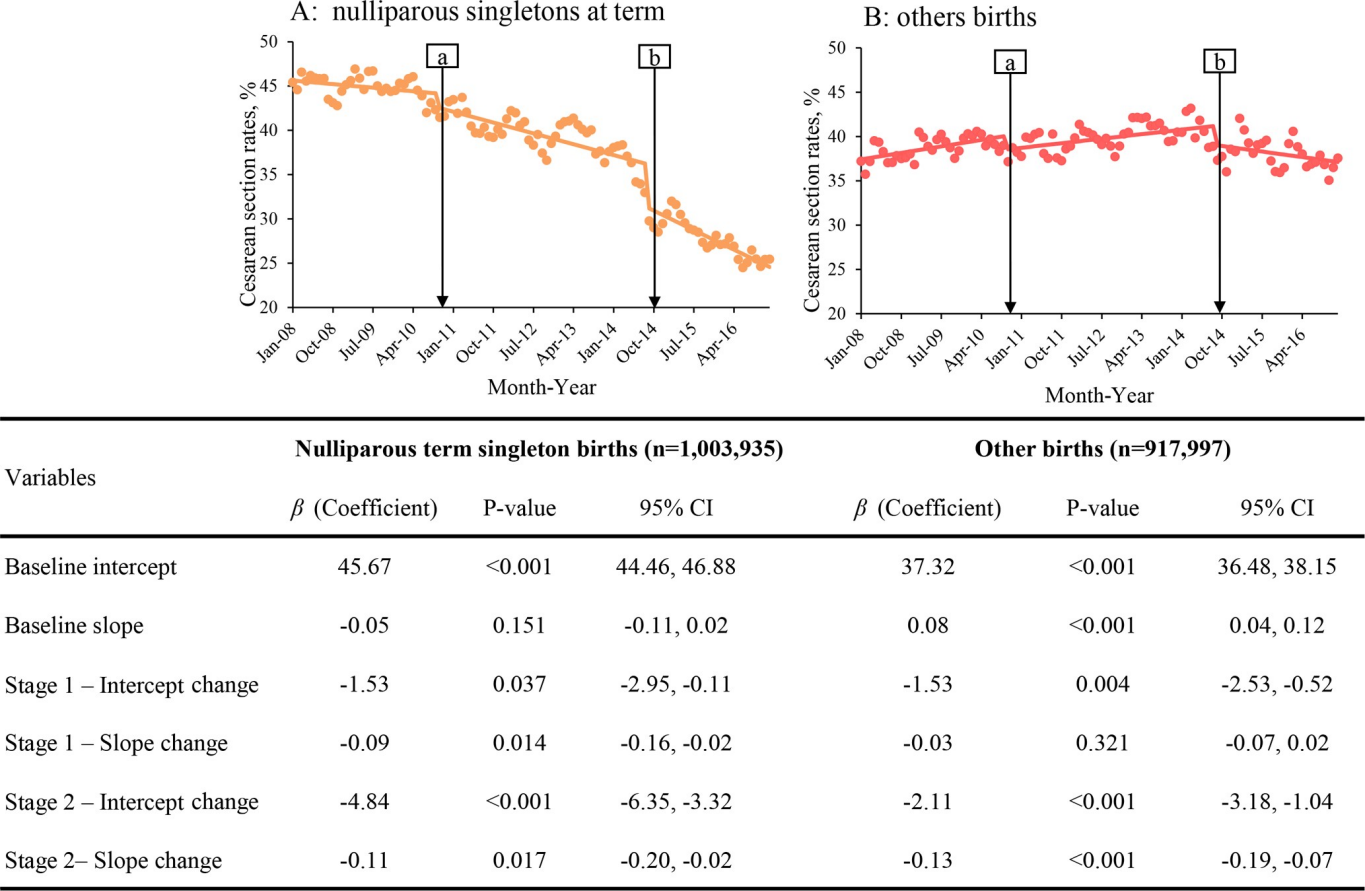
Segmented linear regression models of age-standardized CS rates in nulliparous singletons at term (A) and other births (B) in Guangzhou, China, 2008–2016. Dots indicate true monthly CS rates; solid lines indicate the mean of estimated CS rates per month. Baseline: from January 2008 to September 2010; Stage 1: from October 2010 (Point “a”) to September 2014; Stage 2: from October 2014 (Point “b”) to December 2016. Age-standardized CS rate was calculated by the direct method using 2008 population in each group in the study. Variables listed were modeled along with autoregressive terms by subset models that list the lags in the autoregressive model. Intercept change: change in level compared with the previous stage; slope change: change in trend compared with the previous stage, per month. CS, cesarean section.

Fig 2 shows the results of segmented linear regression analysis for the ASR of CS among nulliparous singletons at term and other births. For nulliparous singletons at term (Fig 2A), ASR was estimated to be 45.7% (95% CI 44.5%–46.9%) at baseline and remained stable (*β* = −0.05; *p* = 0.151). The ASR of CS declined gradually during Stage 1 and Stage 2, with the difference in slope being −0.09 (95% CI −0.16 to −0.02) between Stage 1 and baseline (*p* = 0.014) and −0.11 (95% CI −0.20 to −0.02) between Stage 1 and Stage 2 (*p* = 0.017), respectively. Among other births (Fig 2B), a significant increasing trend in the ASR of CS was observed during the baseline (0.08%/month: 95% CI 0.04%–0.12%, *p* < 0.001). The slope for Stage 1 was of no significant difference compared with baseline, whereas a significantly decreasing slope was observed for Stage 2 compared to Stage 1. Decreases in the ASR of CS were also observed after the introduction of both Stage 1 and Stage 2. Taken together, these indicate an accelerating decreasing trend across 3 stages in the ASR of CS among nulliparous singletons at term, as well as a trend among other births that increased during baseline and Stage 1 and decreased during Stage 2.

Decreasing trends in the CS rate over time were also observed across all age groups (S3 Fig). As high as 93.7%, the ASR of CS among multiparas with prior CS initially increased during baseline, and a declining trend was subsequently observed across Stage 1 and Stage 2 (S3 Table). The analysis excluding the last 3 months in 2016 yielded similar results (S4 Table) as in Fig 2, suggesting that the universal two-child policy (introduced in October 2015) was unlikely to have had immediate effects on the decreasing trend of the CS rate.

**Fig 3:**
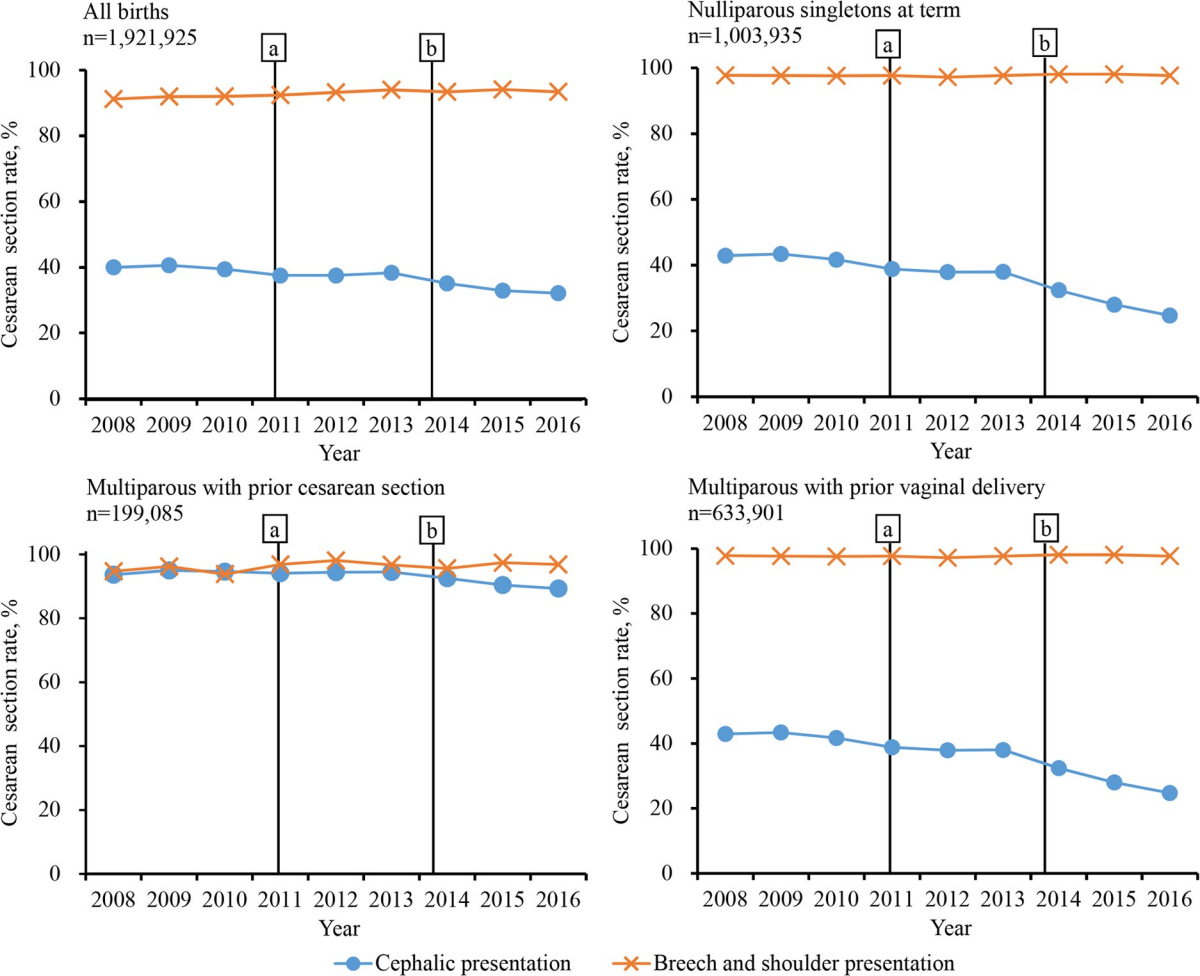
Trends of yearly fetal presentation-specific CS rates among all births and by parity and prior delivery mode in Guangzhou, China, 2008–2016. Baseline: from January 2008 to September 2010; Stage 1: from October 2010 (Point “a”) to September 2014; Stage 2: from October 2014 (Point “b”) to December 2016. CS, cesarean section.

Fig 3 shows the change in annualized fetal presentation-specific CS rates among all births and by parity and prior delivery mode. Consistently high rates of cesarean delivery (>90%) were observed for breech and shoulder presentation at birth between 2008 and 2016. However, the rates were substantially reduced for cephalic presentation, especially among nulliparous singletons at term (42.9% [95% CI 42.4%–43.4%] at baseline, 37.7% [36.9%–38.4%] in Stage 1, and 26.5% [25.7%–27.4%] in Stage 2). Over 90% of women with a prior CS underwent repeated cesarean delivery, but there was a slight decrease of 4.5 percentage points in cesarean delivery for cephalic pregnancies.

### Trends in CS rates on the institutional level

**Fig 4:**
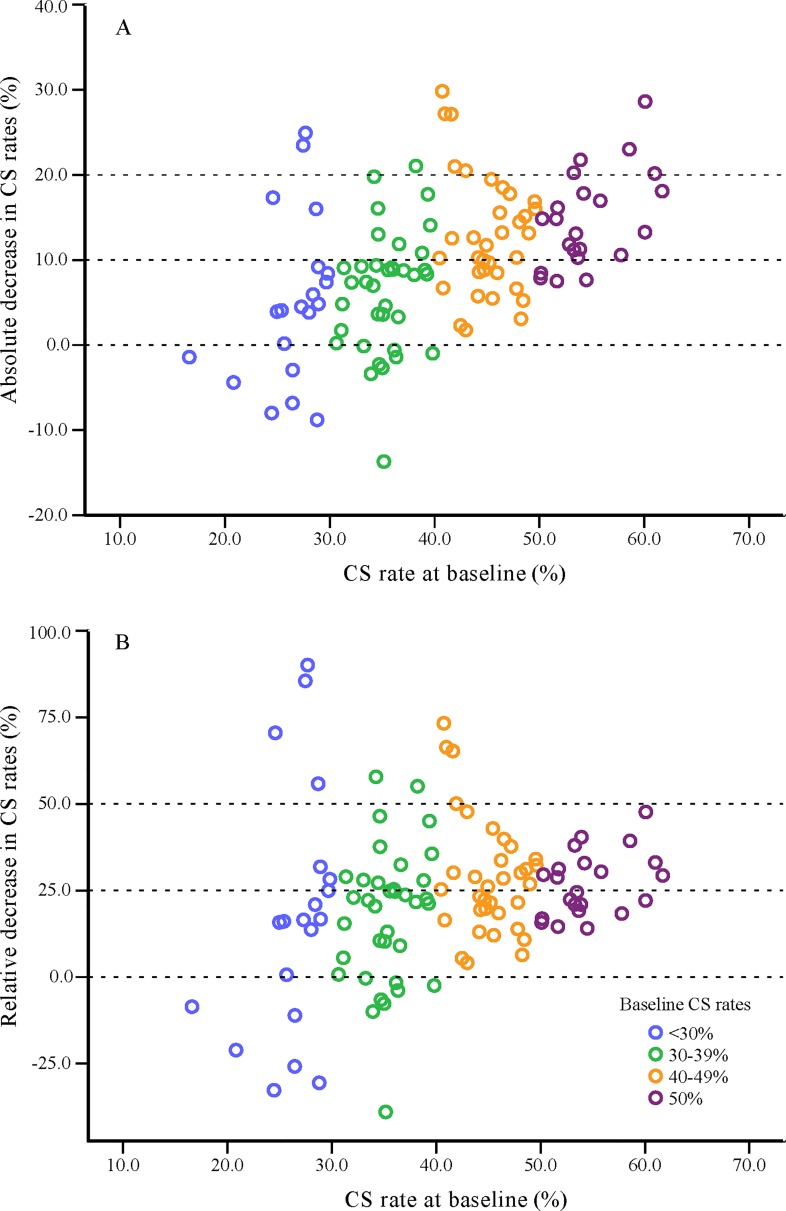
Scatter plot for the absolute decrease in CS rates (A) and the relative decrease in CS rates (B) from 2008 to 2016 among hospitals in Guangzhou grouped by their baseline CS rates. A total of 112 hospitals with more than 100 live births both at baseline and in Stage 2 were included and were grouped by their baseline CS rates. One circle represents one hospital, with blue (*n* = 20), green (*n* = 36), orange (*n* = 34), and purple (*n* = 22) representing baseline CS rates of <30%, 30%–39%, 40%–49%, and ≥50%, respectively. The absolute decrease = the CS rate at baseline − the CS rate in Stage 2; the relative decrease = (the CS rate at baseline − the CS rate in Stage 2) ÷ the CS rate at baseline. CS, cesarean section.

Fig 4 presents the scatter plot for the absolute decrease in CS rates (Fig 4A) and the relative decrease in CS rates (Fig 4B) between baseline and Stage 2 among hospitals grouped by their baseline CS rates (<30%, 30%–39%, 40%–49%, and ≥50%). There were 20, 36, 34, and 22 hospitals, respectively, in each of these 4 groups. The majority (81%) of the hospitals with baseline CS rates of ≥50% had a decline in CS rates of 10% or more in Stage 2, while a quarter of the hospitals with baseline CS rates <30% had increased CS rates over time (Fig 4A). Though the absolute decrease differed across groups (*p* < 0.001), no significant intergroup differences were observed for the relative decrease (*p* = 0.101) (Fig 4B).

### Trends in the maternal and PMR

Accompanying the pronounced reduction in CS rates in 2010–2016 was the significant fall in MMR and PMR (Table 3). Both MMR and PMR decreased in Stage 1 (35% and 19%, respectively) and in Stage 2 (29% and 12%, respectively).

**Table 3:** MMR and PMR by intervention stages in Guangzhou, China, 2008–2016.

	Maternal mortality			Perinatal mortality		
	Deaths (*N*)	Live Births (*N*)	Ratio (1 per 100,000 live births)	Deaths (*N*)	Births (*N*)	Rate (‰)
**Baseline**	84	459,043	18.3	3,368	461,985	7.3
**Stage 1**	107	897,469	11.9	5,347	902,147	5.9
**Stage 2**	47	555,178	8.5	2,921	557,800	5.2
***Z***	−4.3680 (*p* < 0.001)			−13.1420 (*p* < 0.001)		

The *p*-value was obtained from the Cochran-Armitage trend test.

Baseline: January 2008–September 2010; Stage 1: October 2010–September 2014; Stage 2: October 2014–December 2016.

MMR was defined as the number of maternal deaths (during pregnancy or within 42 days of termination of pregnancy, except for accidental deaths) per 100,000 live births; PMR was defined as the number of stillbirths (≥28 weeks of gestation) and early neonatal deaths (0–6 days) per 1,000 births.

**Abbreviations:** MMR, maternal mortality ratio; PMR, perinatal mortality rate

## Discussion

### Main findings

The present study examined changes of overall CS rate over the period of a 6-year two-stage intervention in Guangzhou, China. Cesarean delivery rate fell from 42% before intervention to 40% in Stage 1 and to 35% in Stage 2. The decline was prominent among nulliparous singletons at term. There was no observable impact of the universal two-child policy on CS rate. Nevertheless, despite the overall decline, more than 90% of women with a prior cesarean went on to a repeated CS. Our study has provided evidence to support the use of similar multifaceted intervention strategies to improve public health in China and elsewhere.

### Strengths and limitations

An important strength of this study is the use of high-quality city-wide surveillance data with 99% coverage of all deliveries over 9 years to examine the changes in cesarean delivery rates before and after the implementation of strategies. However, our study has several limitations. First, as the intervention was implemented across all 12 districts in Guangzhou at the same time, we were not able to compare CS rates with a parallel control group in this natural experiment and had to rely on the pre-intervention period (January 1, 2008–September 30, 2010) as the baseline for a before-and-after comparison. However, Guangzhou seemed to have a higher relative decrease in yearly average CS rates from 2012 to 2016 when comparing with a national sample of 438 hospitals from 326 districts and counties (12% versus 9%) [19], suggesting that the intervention might have exerted an impact. Second, we were only able to calculate overall CS rate, because our data did not allow us to differentiate between necessary and unnecessary CS. Objective and detailed review of the 1.9 million births to obtain information on the underlying circumstances and indications is not feasible. However, our findings did provide some indirect evidence supporting the decrease of the unnecessary CS rate. CSs for women with cephalic pregnancy are likely to be unnecessary, whereas for those with noncephalic pregnancy, a CS would be necessary to reduce maternal and infant mortality. Our study showed that CS rate for cephalic presentation greatly decreased over time, but a similar trend was not observed for breech and shoulder presentation. In addition, both MMR and PMR also declined with CS rate. All these together suggest that the reduction in unnecessary CS was largely responsible for the observed decline in overall CS rate. Third, the GPHCDSS was set up in 2000 for audit and not research purposes. Information on pregnant women’s knowledge of and attitude towards CS [32–34] was not recorded in the GPHCDSS, which might have changed during the intervention period and might have had an impact on the CS rate regardless of the intervention [23]. Fourth, some hospitals had implemented certain interventions even before Stage 1, such as dissemination of the advantages of vaginal delivery and training for obstetricians, which might have diluted the observed decline between baseline and Stage 1. Fifth, prior CS was defined on the basis of parity and the presence of uterine scarring, which could be prone to misclassification due to previous surgical operations in the uterus. Lastly, given the observational nature of the present study and the unmeasured changes of the population over the study duration, causality cannot be inferred.

### Interpretation

In the present study, CS rates of nulliparas were originally higher than those of all births at baseline but were 5.9% lower in Stage 2. Thus, we speculate that nulliparous women may be more likely to benefit from the quality improvement initiatives [35,36]. Also, given the high repeated CS rate for multiparous women, we believe that future interventions should target nulliparas primarily.

CS rates among childbirths with cephalic presentation declined substantially, especially among nulliparous term singleton births, but remained high among women with prior CS, consistent with findings in another nationwide study in China [19]. Hospital-based data across the country have shown that, in 2016, rates of CS in multiparous women with and without uterine scarring were 90.4% and 18.5%, respectively [19]. A Chinese expert consensus published in 2014 introduced 15 indications for CS, both applicable to nulliparous and multiparous women, where a scarred uterine with ≥2 previous cesarean deliveries is indicative for CS [37].

Previous evidence suggests that, when compared with elective repeated cesarean delivery, trial of labor after cesarean delivery (TOLAC) may be associated with similar or lower risks of maternal and neonatal morbidity in women with a likelihood of achieving vaginal birth after cesarean delivery (VBAC) above a threshold of 60% to 70% [38,39]. A 92.6% rate of repeat CS was observed in the present study, even higher than that reported among 871,636 deliveries by Chinese women with a previous CS during 2012–2016 (90.2%) [40]. Consultation on VBAC is supposed to be provided to pregnant women with a prior cesarean delivery who are eligible for TOLAC. However, there are currently no generally accepted national guidelines and prediction models on VBAC in China. While an expert consensus on indications and contraindications of trial of labor after CS was issued by the Chinese Society of Obstetrics and Gynecology in 2016 [41], there was little guidance on the management of perinatal adverse outcomes, and the evidence base for the consensus was insufficient [40]. National guidelines and clinical support are called for to provide a functioning environment for TOLAC in China.

The family planning policy in China has been discussed in detail by Zeng and Hesketh [42]. Until its abolishment, a strict one-child policy had been in place in China for nearly 30 years since 1979. In 2007, couples who were both only children were allowed to have a second child. In 2014, the policy was further relaxed to permit families to have two children if one of the parents was an only child. Subsequently, a universal two-child policy was implemented nationally in October 2015. The long-term impact of the universal two-child policy on CS rate is complex, and the direction and magnitude of the change in CS rate is difficult to predict. On one hand, the change in family planning policy may result in a large number of multiparas with a prior history of CS and an advanced maternal age, whose attempt to have a second child was previously forbidden. Although the advancement of maternal age is not an indicator for CS, it is associated with weakening of the myometrium, reduction in the number of oxytocin receptors, the lower clinical threshold for obstetric interventions, and increased rates of maternal systemic diseases and obstetric complications, all of which contribute to an increased risk of CS [43]. On the other hand, however, nulliparas are more likely to choose vaginal birth in consideration of future pregnancies [44], which may reduce cesarean delivery on maternal request. While our findings suggest that the universal two-child policy did not seem to have influenced CS rate in the first 14 months of the new policy in Guangzhou, we do not have longer-term data to rule out any delayed effect, which may not be evident until years later.

Preference for mode of delivery among the Chinese population has been suggested to be affected by various factors, as shown by a recent systematic review that CS was preferred due to fear of pain during labor and of perceived risks to the baby associated with vaginal delivery. In addition, some factors unique to mainland China, such as disincentivizing interactions with the healthcare system, tensions and mistrust between doctors and patients, and superstitions about certain birth dates being auspicious also influence women’s preference and doctors’ decision, making them more likely to have elective CS [23,24]. As it is more difficult to change the culture, the intervention focused on health education that aimed to increase knowledge and awareness of the disadvantages of CS and the advantages of vaginal delivery through an intensive publicity campaign. Hospitals with a higher baseline CS rate had a greater absolute decrease compared with the baseline. Given the large number of annual births in Guangzhou, the hospitals of high baseline CS rates could be considered the targeted hospitals for reducing CS rates. We estimated that a reduction of a percentage point in CS rate would result in approximately 2,500 less births from CS each year based on the number of births in 2016. Hospitals with different baseline CS rates showed similar relative decreases after the intervention, which illustrated the universality and generalizability of this program.

Along with the substantial reduction in the CS rate, a decrease in maternal and perinatal mortality was also observed in Guangzhou. It was reported that in 2008, 4 large cities in China—Beijing, Tianjin, Hangzhou, and Shanghai—had relatively low maternal and perinatal mortality (8–11 per 100,000 live births for MMR and 3–5 per 1,000 births for PMR). From 2008 to 2014, perinatal mortality decreased in the former two cities and remained stable in the latter two, while maternal mortality stayed unchanged in all 4 of these cities [18]. Our data show that the annual MMR and PMR in Guangzhou in 2008 were 22 per 100,000 live births and 7 per 1,000 births, respectively, higher than that reported in the 4 cites. Differences in characteristics of pregnant women and in specific local maternal and neonatal healthcare intervention strategies between cities might result in these different baseline and temporal trends of MMR and PMR. The intervention reported here was a part of the municipality-wide Action Plan for Safe Motherhood and Infancy, and therefore the impact of CS-rate decline on MMR and PMR cannot be concluded in isolation. However, our findings did provide some evidence that the CS rate can be decreased by a specific intervention without impacting maternal and perinatal mortality.

In conclusion, an apparent decline in CS rate was observed after the implementation of a two-stage intervention in Guangzhou, the largest city in southern China. The decline was more evident among nulliparous women who delivered term singletons. Given the increasing maternal age in urban China, the relaxation of the one-child policy, and the high CS rate among women with prior CS, future efforts to control CS rates in China could be challenging. Guangzhou’s experience may have the following implications for future policy-making to reduce CS rates in other similar areas with high rates of CS: (1) social consensus for reducing CS rates should be prioritized without setting a specific target rate for the reduction, and (2) composite interventions should be considered to improve overall maternal and perinatal health instead of targeting CS alone.

## Supporting information

S1 Checklist STROBE and RECORD checklists. (DOCX)

S1 File Analysis plan. (DOCX)

S1 Fig The networks of the referral system and of the maternal and child healthcare system in Guangzhou. (TIF)

S2 Fig Change of the proportion of parturient women by maternal age (panel A) and by parity and prior delivery mode (panel B) in Guangzhou, China, 2008–2016.
(TIF)

S3 Fig **Segmented linear regression models of CS rates by maternal age in all births (panel A), nulliparous term singleton births (panel B), and other births (panel C), in Guangzhou, China, 2008–2016.** Dashed lines indicate true monthly CS rates; solid lines indicate the mean of estimated value by segmented regression models. Baseline: from January 2008 to September 2010; Stage 1: from October 2010 (Point “a”) to September 2014; Stage 2: from October 2014 (Point “b”) to December. EST, the mean of estimated value by segmented regression model; OBS, observed monthly CS rates.
(TIF)

S1 Table Perinatal characteristics and CS rates by intervention stages among nulliparous singletons at term in Guangzhou, China, 2008–2016 (*n* = 1,003,935). (XLSX)

S2 Table Perinatal characteristics and CS rates by intervention stages among others births in Guangzhou, China, 2008–2016 (*n* = 917,997). (XLSX)

S3 Table Segmented linear regression models of age-standardized CS rates by prior delivery mode, in Guangzhou, China, 2008–2016. (XLSX)

S4 Table Sensitivity analysis of age-standardized CS rates by segmented linear regression models in all births, nulliparous term singleton births, and all other births, in Guangzhou, China, 2008–2016, excluding the last 3 months in 2016. (XLSX)

## Abbreviations

ASR: age-standardized rate
CS: cesarean section
GPHCDSS: Guangzhou Perinatal Health Care and Delivery Surveillance System
MMR: maternal mortality ratio
PMR: perinatal mortality rate
RECORD: REporting of studies Conducted using Observational Routinely-collected health Data
TOLAC: trial of labor after cesarean delivery
VBAC: vaginal birth after cesarean delivery
WHO: World Health Organization
