# Supplementary material for: Effect of a two-stage intervention package on the cesarean section rate in Guangzhou, China: A before-and-after study
Source: PLoS Med. 2019 Jul 8;16(7):e1002846. doi: 10.1371/journal.pmed.1002846 (PMC6613675; doi:10.1371/journal.pmed.1002846)
Supplement: S1 File — (DOCX) [file pmed.1002846.s002.docx]

**Effectiveness of a Two-stage Intervention Package to Reduce the Cesarean Section Rate in A Large City in China: A Before-and-After Study**

**Analysis plan**

**June 2017**

1. Research Question

·What are the trends of Cesarean section rate (CS rate), maternal mortality ratio (MMR) and perinatal mortality rate (PMR) during the Two-stage intervention conducting period in Guangzhou?

·What are the effects of Two-Stage Intervention at the end of the intervention plan?

1. Background

The cesarean section (CS) rate has risen globally during the last two decades.(1, 2) Although CS is being essential and lifesaving with the correct indication, it is used unnecessarily on non-emergency conditions and leads to greater financial costs without proven benefit yet increasing risks for maternal morbidity and mortality.(3-9) In addition, CS has been associated with elevated risks of adverse maternal and perinatal outcomes in future pregnancies, even maternal and intra-uterine fatal death.(10-13)

The risk for adverse outcomes from the increasing CS rate has prompted the Health Commission of Guangzhou Municipality to investigate strategies to reduce CS rates and to improve the quality of intrapartum care.(14) Several Studies identified a number of strategies shown to be effective in a range of settings.(15-17) Based on the previous findings, a two-stage intervention package was developed by the Health Commission of Guangzhou Municipality, and subsequently implemented from 2010 to 2016, as an important part of the Action Plan for Safe Motherhood and Infancy aiming to control and reduce maternal and infant mortality in Guangzhou.

It will be the first time that the effects of the two-stage intervention package on reducing CS rate in Guangzhou be analyzed and investigated. The evaluation on the application and benefits of the package will provide evidence to the Health Commission of Guangzhou Municipality and its health services in policy development and planning in Guangzhou.

**Aims of the study:**

·To describe trend of CS rate before and after the two-stage intervention package

·To evaluate the effects of the two-stage intervention package on controlling and reducing maternal and infant mortality in Guangzhou.

1. Methods

The study will be a population-based retrospective evaluation on a two-stage intervention package with quasi-experimental design by the municipal government for reducing the CS rate.

3.1 Data source and study population

We will define the pre-intervention period between January 1, 2018 and September 30, 2010 as baseline (total 33 weeks); the period between October 1, 2010 and September 30, 2014 as the first stage of intervention (total 48 weeks); and October 1, 2014 and December 31, 2016 as the second stage of intervention (total 28 weeks). Birth data will be obtained from Guangzhou Perinatal Health Care and Delivery Surveillance System (GPHCDSS). Data on maternal deaths will collect through the Maternal and Child Health Data Reporting Network in China.

3.1.1 Data source will be analyzed：

**Exposure data source**

The births delivered at gestational age of ≥28 weeks will be included. The information on maternal age, parity, gestational age, birth weight, and mode and outcomes of delivery will be collected from GPHCDSS. Observations with extreme values (e.g. parity and gravidity above the 99.98 percentile or women aged <14 years) or missing gestational age information will be excluded.

**Exposure data sources definition:**

**·Maternal age**

<25, 25-29, 30-34, and ≥35 years

**·Parity**

Nulliparous, multiparous with prior vaginal delivery, multiparous with prior CS

**·Gestational age**

Preterm (<37 weeks), term (37-42 weeks) and post-term (>42 weeks)

**·Birth weight**

Low birth weight (<2500 g), normal birth weight (2500-3999 g) and macrosomia (≥4000 g)

**·Mode**

[Natural](javascript:;) labor, cesarean delivery

**·Delivery outcomes**

Maternal death, perinatal death

**Outcome data source**

The outcomes will include CS rate, MMR and PMR.

The primary outcome will be CS rate, defined as the number of caesarean deliveries per 100 births. CS rate will be calculated monthly, six month moving average (M.V.6) and each intervention stage (baseline, the first stage and the second stage).

The secondary outcomes will be the MMR, defined as the number of maternal deaths (during pregnancy or within 42 days of termination of pregnancy, except for accidental deaths) per 100 000 live births and the PMR, defined as the number of stillbirths and early neonatal deaths per 1000 births.

3.2 Statistical analysis

Average CS rate will be described among all births and cross-tabulated with maternal and perinatal characteristics, parity, gestational age, birth weight for each of the three stages, respectively. Original checking from YMY: Average CS rate will be described in each intervention stage account for maternal and perinatal characteristics, parity, gestational age, birth weight.

The Cochran-Armitage test will be used to examine the changing trends of monthly CS rates, MMR and PMR. To understand the changing trends in CS rates over time on the institutional level, Hospitals that remained in the midwifery system in Guangzhou during 2008-2016, with more than 100 live births at both baseline and in Stage 2, will be grouped according to their baseline CS rates (<30%, 30-39%, 40-49%, and ≥ 50%), and both the absolute and the relative decrease in the CS rate of individual hospitals will be depicted on a scatter plot. One-way analysis of variance will be used to compare the decrease in CS rates across different groups of hospitals. To clarify the effect of fetal presentation on cesarean deliveries, we will analyze breech and shoulder presentation and head presentation separately stratifying for parity.

**Software**

The analysis will be performed by using SAS 9.3 (SAS Institute Inc., Cary, NC, USA) and SPSS 23.0 (SPSS Inc., Chicago, Ill., USA). All P values will be two-sided, and P<0.05 will be considered to indicate statistical significance.

1. Counseling Team

Mr. Pingyan Chen|School of Public Health, Southern Medical University

Skills and expertise: Biostatistics

Dr. Linhong Wang|National Cnetre for Women and Children’s Health, Chinese centre for disease control and prevention

Skills and expertise: Women’s health and reproductive health

Dr. Zilian Wang|The First Affiliated Hospital, Sun Yat-Sun University

Skills and expertise: perinatal medicine

Dr. Xiaomin Xiao|The First Affiliated Hospital of Jinan University

Skills and expertise: perinatal medicine and assisted reproduction

Dr. Jinxin Zhang|School of Public Health, Sun Yat-Sun University

Skills and expertise: Biostatistics

1. Project stages

**Stage 1: Project design**

·Determine project analysis plan

·Decide methodology and process

·Complete literature review

**Stage2: Data preparation**

·Data access

·Data cleansing

**Stage 3: Data analysis**

**Stage 4: Paper preparation**

·Complete draft paper

**Refer to Appendix 1 for Project Grantt Chart**

**Appendix 1 Project Grantt Chart**

| Year | **2017** | | | | | | | **2018** | | | | | | | | | | | |
| --- | --- | --- | --- | --- | --- | --- | --- | --- | --- | --- | --- | --- | --- | --- | --- | --- | --- | --- | --- |
| Months | Jun | Jul | Aug | Sep | Oct | Nov | Dec | Jan | Feb | Mar | Apr | May | Jun | Jul | Aug | Sep | Oct | Nov | Dec |
| Stage1 |  |  |  |  |  |  |  |  |  |  |  |  |  |  |  |  |  |  |  |
| Analysis plan |  |  |  |  |  |  |  |  |  |  |  |  |  |  |  |  |  |  |  |
| Methodology |  |  |  |  |  |  |  |  |  |  |  |  |  |  |  |  |  |  |  |
| literature review |  |  |  |  |  |  |  |  |  |  |  |  |  |  |  |  |  |  |  |
|  | | | | | | | | | | | | | | | | | | | |
| Stage2 |  |  |  |  |  |  |  |  |  |  |  |  |  |  |  |  |  |  |  |
| Data access |  |  |  |  |  |  |  |  |  |  |  |  |  |  |  |  |  |  |  |
| Data cleansing |  |  |  |  |  |  |  |  |  |  |  |  |  |  |  |  |  |  |  |
|  | | | | | | | | | | | | | | | | | | | |
| Stage3 |  |  |  |  |  |  |  |  |  |  |  |  |  |  |  |  |  |  |  |
| Data analysis |  |  |  |  |  |  |  |  |  |  |  |  |  |  |  |  |  |  |  |
|  | | | | | | | | | | | | | | | | | | | |
| Stage 4 |  |  |  |  |  |  |  |  |  |  |  |  |  |  |  |  |  |  |  |
| Complete draft paper |  |  |  |  |  |  |  |  |  |  |  |  |  |  |  |  |  |  |  |

Duration

Sub-duration

**Reference**

1. Molina G, Weiser TG, Lipsitz SR, Esquivel MM, Uribe-Leitz T, Azad T, et al. Relationship Between Cesarean Delivery Rate and Maternal and Neonatal Mortality. Jama. 2015;314(21):2263-70.

2. Chaillet N, Dumont A, Abrahamowicz M, Pasquier JC, Audibert F, Monnier P, et al. A cluster-randomized trial to reduce cesarean delivery rates in Quebec. N Engl J Med. 2015;372(18):1710-21.

3. Li H, Ye R, Pei L, Ren A, Zheng X, Liu J. Caesarean delivery, caesarean delivery on maternal request and childhood overweight: a Chinese birth cohort study of 181 380 children. Pediatr Obes. 2014;9(1):10-6.

4. O'Neill SM, Agerbo E, Kenny LC, Henriksen TB, Kearney PM, Greene RA, et al. Cesarean section and rate of subsequent stillbirth, miscarriage, and ectopic pregnancy: a Danish register-based cohort study. PLoS medicine. 2014;11(7):e1001670.

5. Blustein J, Liu J. Time to consider the risks of caesarean delivery for long term child health. BMJ. 2015;350:h2410.

6. Camara R, Burla M, Ferrari J, Lima L, Amim JJ, Braga A, et al. Cesarean section by maternal request. Revista do Colegio Brasileiro de Cirurgioes. 2016;43(4):301-10.

7. Wu X, Huang K, Chen Y. Cesarean section may increase the risk of both overweight and obesity in preschool children. BMC pregnancy and childbirth. 2016;16(1).

8. Caceres IA, Arcaya M, Declercq E, Belanoff CM, Janakiraman V, Cohen B, et al. Hospital differences in cesarean deliveries in Massachusetts (US) 2004-2006: the case against case-mix artifact. PloS one. 2013;8(3):e57817.

9. American College of O, Gynecologists, Society for Maternal-Fetal M, Caughey AB, Cahill AG, Guise JM, et al. Safe prevention of the primary cesarean delivery. American journal of obstetrics and gynecology. 2014;210(3):179-93.

10. Landon MB, Hauth JC, Leveno KJ, Spong CY, Leindecker S, Varner MW, et al. Maternal and perinatal outcomes associated with a trial of labor after prior cesarean delivery. N Engl J Med. 2004;351(25):2581-9.

11. Solheim KN, Esakoff TF, Little SE, Cheng YW, Sparks TN, Caughey AB. The effect of cesarean delivery rates on the future incidence of placenta previa, placenta accreta, and maternal mortality. The journal of maternal-fetal & neonatal medicine : the official journal of the European Association of Perinatal Medicine, the Federation of Asia and Oceania Perinatal Societies, the International Society of Perinatal Obstet. 2011;24(11):1341-6.

12. O'Neill SM, Kearney PM, Kenny LC, Khashan AS, Henriksen TB, Lutomski JE, et al. Caesarean delivery and subsequent stillbirth or miscarriage: systematic review and meta-analysis. PloS one. 2013;8(1):e54588.

13. Smith GC, Pell JP, Cameron AD, Dobbie R. Risk of perinatal death associated with labor after previous cesarean delivery in uncomplicated term pregnancies. Jama. 2002;287(20):2684-90.

14. Li HT, Luo S, Trasande L, Hellerstein S, Kang C, Li JX, et al. Geographic Variations and Temporal Trends in Cesarean Delivery Rates in China, 2008-2014. Jama. 2017;317(1):69-76.

15. Chaillet N, Dumont A. Evidence-based strategies for reducing cesarean section rates: a meta-analysis. Birth. 2007;34(1):53-64.

16. Sheikh L, Tehseen S, Gowani SA, Bhurgri H, Rizvi JH, Kagazwala S. Reducing the rate of primary caesarean sections--an audit. JPMA The Journal of the Pakistan Medical Association. 2008;58(8):444-8.

17. Taljaard M, Donner A, Villar J, Wojdyla D, Faundes A, Zavaleta N, et al. Understanding the factors associated with differences in caesarean section rates at hospital level: the case of Latin America. Paediatric and Perinatal Epidemiology. 2009;23(6):574-81.
