# Supplementary figures and images for: Effect of a two-stage intervention package on the cesarean section rate in Guangzhou, China: A before-and-after study
Source: PLoS Med. 2019 Jul 8;16(7):e1002846. doi: 10.1371/journal.pmed.1002846 (PMC6613675; doi:10.1371/journal.pmed.1002846)

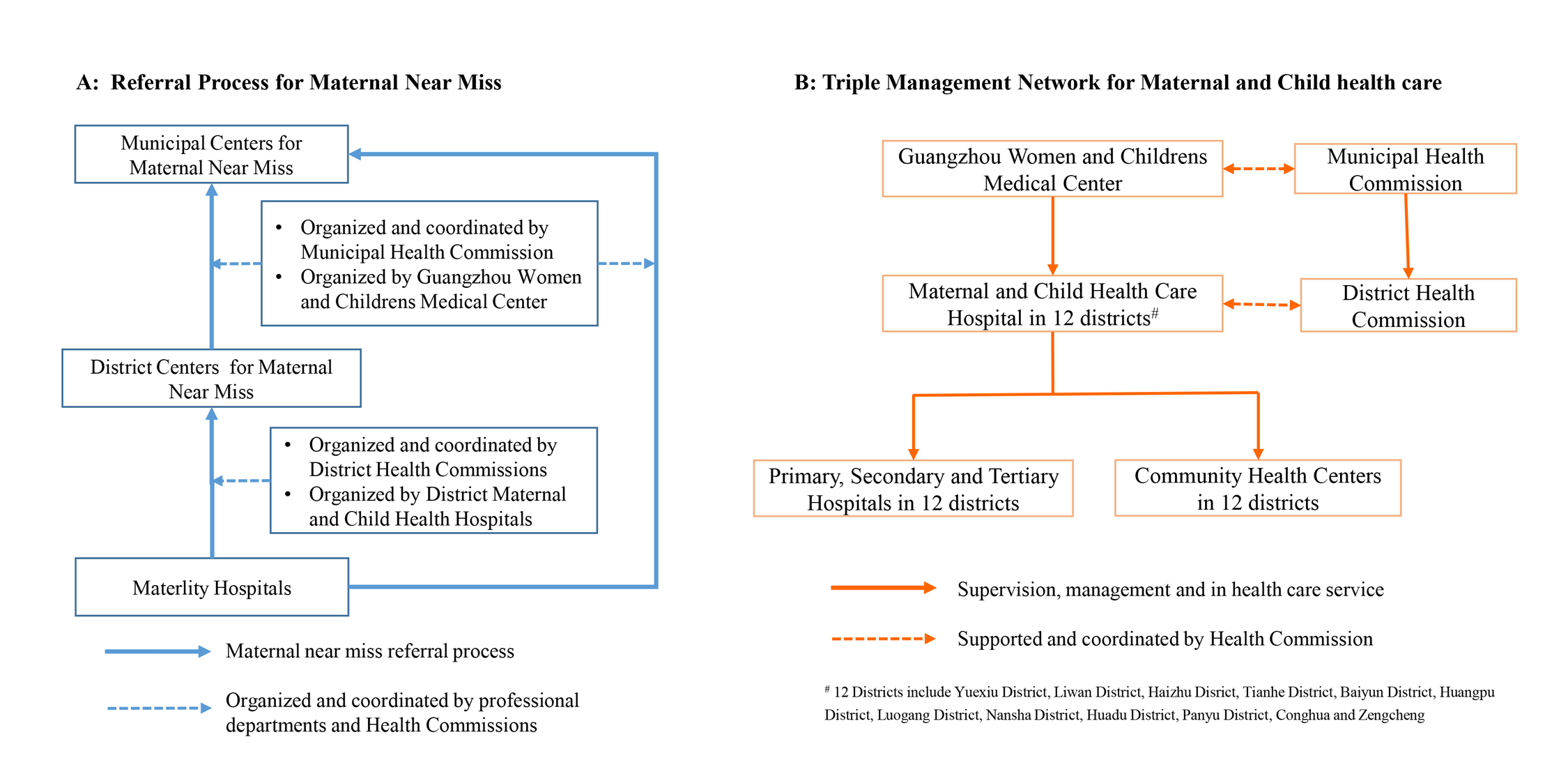

Supplement: S1 Fig — (TIF) [file pmed.1002846.s003.tif]

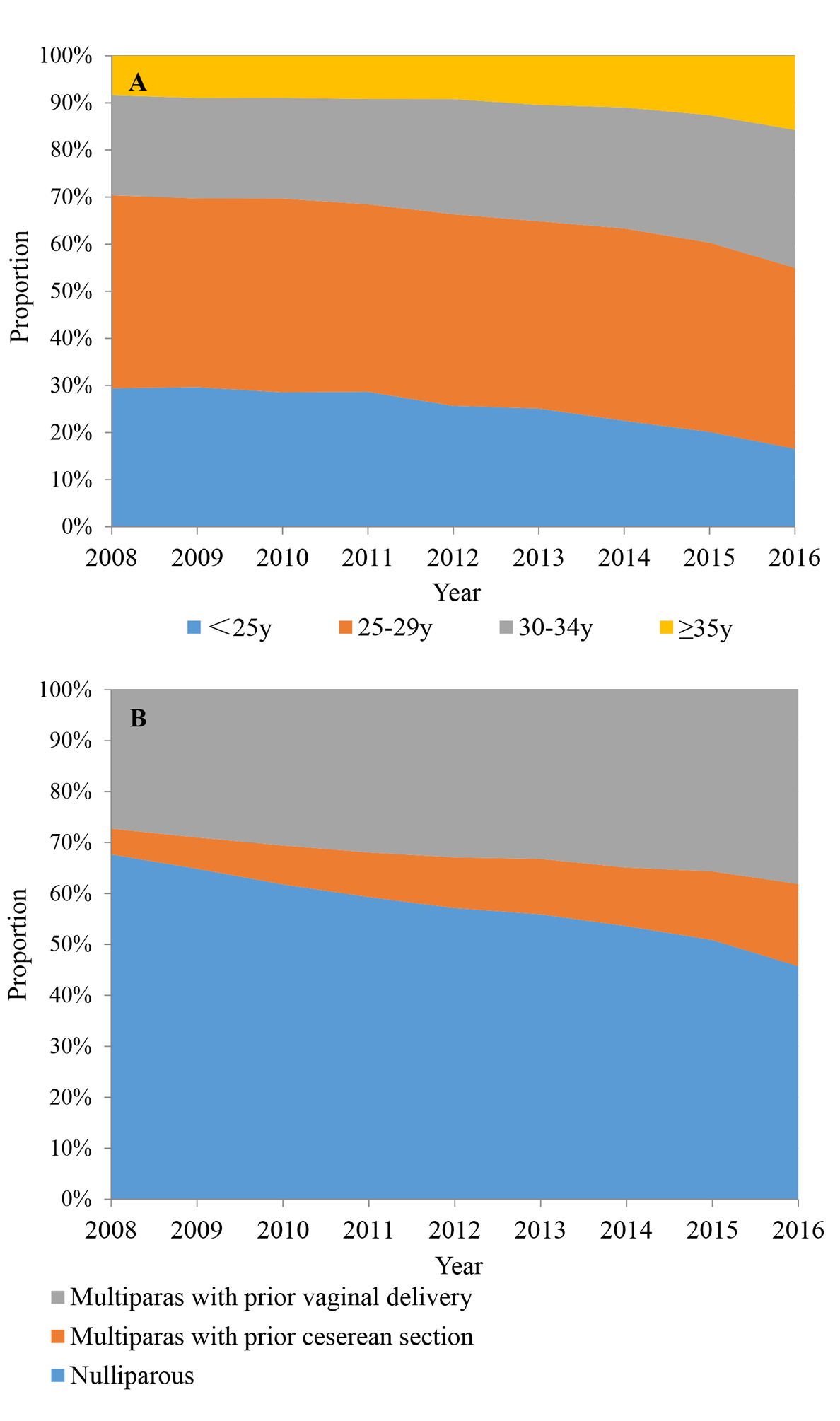

Supplement: S2 Fig — Change of the proportion of parturient women by maternal age (panel A) and by parity and prior delivery mode (panel B) in Guangzhou, China, 2008–2016. (TIF) [file pmed.1002846.s004.tif]

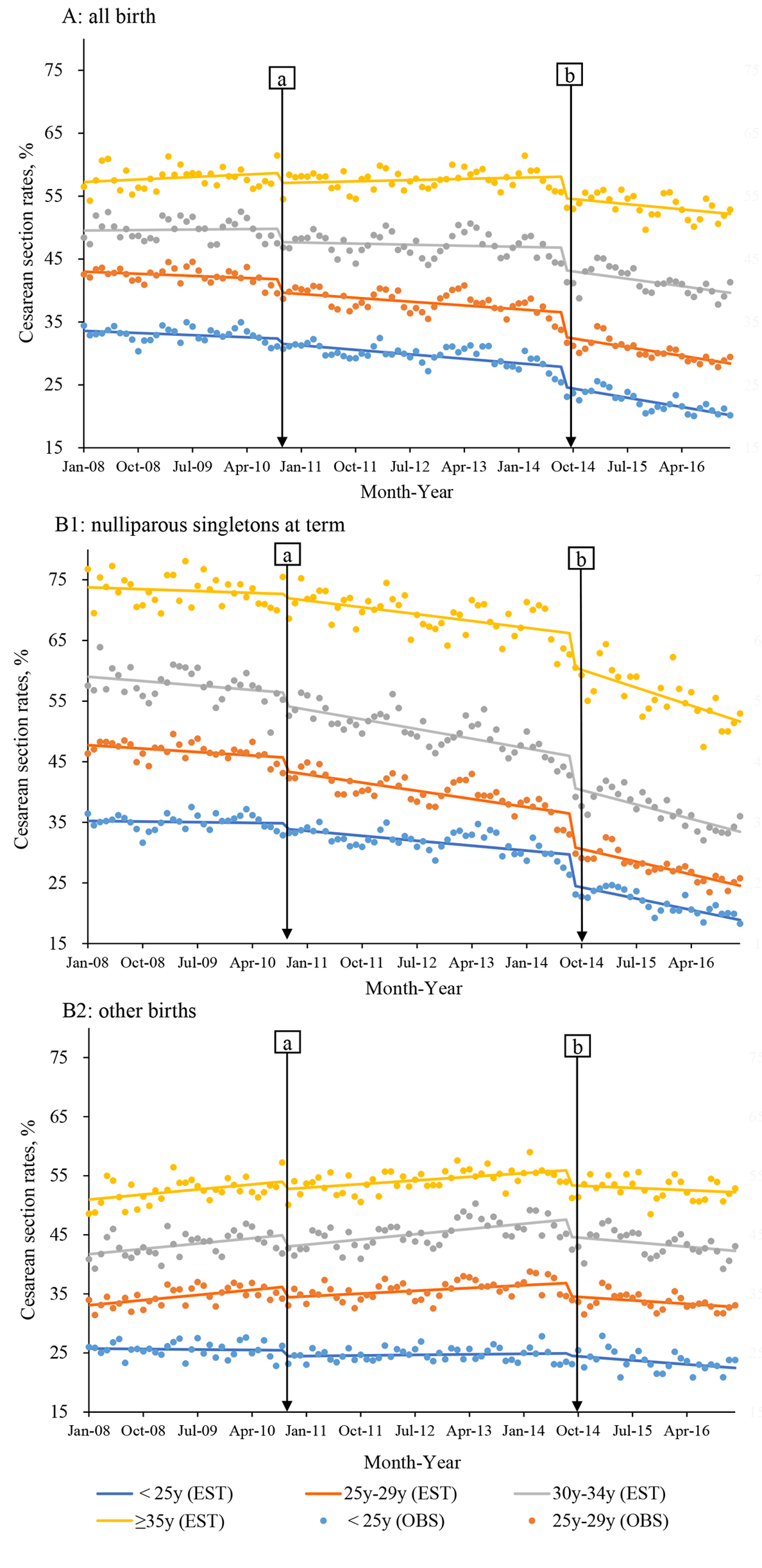

Supplement: S3 Fig — Segmented linear regression models of CS rates by maternal age in all births (panel A), nulliparous term singleton births (panel B), and other births (panel C), in Guangzhou, China, 2008–2016. Dashed lines indicate true monthly CS rates; solid lines indicate the mean of estimated value by segmented regression models. Baseline: from January 2008 to September 2010; Stage 1: from October 2010 (Point “a”) to September 2014; Stage 2: from October 2014 (Point “b”) to December. EST, the mean of estimated value by segmented regression model; OBS, observed monthly CS rates. (TIF) [file pmed.1002846.s005.tif]
